# Case Report: Multifocal Epithelial Hyperplasia in Two Yemeni Patients With Khat Chewing Habits

**DOI:** 10.1155/crid/8090225

**Published:** 2025-11-28

**Authors:** Layla Hafed, Munia Mahmood Shamsheer, Islam Alobaidy, Eman Abdullah S. Al-Salami, Fathia Gazem S. Awadhi, Hind Arhab, Aisha A. H. Al-Jamaei

**Affiliations:** ^1^Department of Oral Medicine and Diagnostic Science, Faculty of Dentistry, Saba University, Sana'a, Yemen; ^2^Oral and Maxillofacial Pathology, Oromed Labs, Cairo, Egypt; ^3^Department of Oral Medicine, Periodontology, and Radiology, Faculty of Dentistry, Sana'a University, Sana'a, Yemen; ^4^Histopathology and Tumors Department, Al-Mamoon Diagnostic Medical Center, Sana'a, Yemen; ^5^Dermatology Department, Al-Mamoon Diagnostic Medical Center, Sana'a, Yemen; ^6^Department of Oral Surgery and Oral Medicine, Faculty of Dentistry, Al-Razi University, Sana'a, Yemen

## Abstract

Multifocal epithelial hyperplasia (MEH) represents a benign condition associated with human papillomavirus. This condition is notably uncommon and primarily affects specific demographic groups, particularly children and adolescents. Within Yemen, similar to other Asian regions, the condition remains relatively uncommon. The lesions typically manifest intraorally on the labial and buccal mucosa as well as the tongue. In this report, we present two cases, where MEH was observed on the vermillion surface of the lips in adult patients. Several possible risk factors were identified in these cases, including socioeconomic disadvantage, khat chewing, and liver dysfunction. These findings emphasize the importance of considering MEH in the differential diagnosis of papillary lesions affecting the vermillion surfaces of the lips.

## 1. Introduction

Multifocal epithelial hyperplasia (MEH), or Heck's disease, was initially documented by Dr. Heck et al. in 1965 [1]. Subsequently, 11 additional cases were documented within the same year [2]. The condition demonstrates a higher prevalence among specific ethnic populations, notably Native Americans across Central, South, and North America, as well as the Inuit and Yupik of indigenous ancestry. In contrast, it appears less frequently among individuals of European and African descent and is relatively uncommon in those of Asian heritage [3]. The concentration of this benign, clinically distinct condition within specific lineages and geographic regions was previously attributed to environmental and genetic factors; however, its etiology is primarily associated with HPV, particularly Subtypes 13 and 32 [4, 5]. Research has also documented cross-reactivity with other HPV subtypes [3, 6, 7]. Regarding genetic susceptibility, human lymphocytic antigen (HLA-DR4, DRB10404) appears to play a significant role [8]. Studies indicate that Subtype 32 predominantly affects the elderly demographic, while Subtype 13 manifests in both younger and older patient populations [9].

In general terms, the condition demonstrates a predilection for children and adolescents, with a higher incidence observed in female patients [10]. These HPV subtypes exhibit affinity for specific mucosal surfaces, manifesting in both keratinized and nonkeratinized mucosa. The lower labial mucosa represents the most common site of occurrence, followed by the upper labial mucosa, buccal mucosa, and tongue [11]. Other locations are rarely affected, including the gingiva and anterior faucial pillars [12]. Clinical presentation typically features multiple, size-limited papules that match the color of the surrounding mucosa [2]. Spontaneous resolution may occur within approximately 18 months; however, certain lesions may persist for extended periods [13]. It is noteworthy that no studies have identified precancerous potential in these lesions despite their HPV etiology [8].

Khat consumption is common in Yemen and several African countries [14, 15]. Studies show this practice significantly affects both oral [16] and overall health [17]. In the oral cavity, it can lead to various conditions including white lesions [18], erythroplakia [19], and in some cases, squamous cell carcinoma (SCC) [20], along with other health issues [16, 21].

This paper presents two cases of MEH in adult Yemeni patients with documented khat chewing habits. One case tested positive for HPV 16 and was confined to vermillion surfaces, while the other tested negative and involved both vermillion surfaces and intraoral regions.

## 2. Case 1

**Figure 1:**
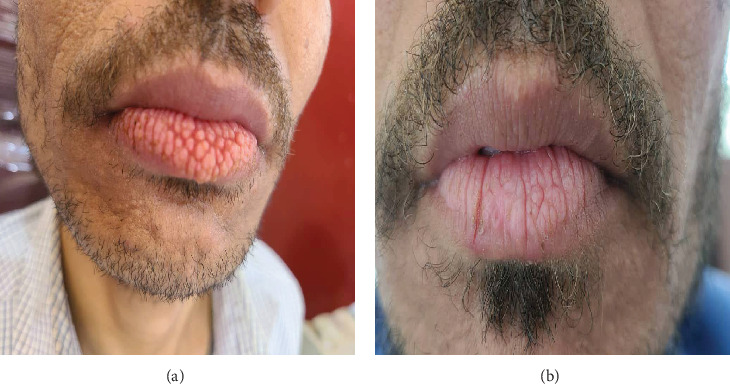
Clinical photograph of Case 1 showing (a) patient presenting with multiple papillary lesions on the vermillion surface of the lower lip. (b) Following the cessation of khat chewing, the patient exhibited notable regression of the lesions.

A 34-year-old male engineer presented at Sana'a University's Oral Medicine Outpatient Clinic on February 11, 2025, with concerns regarding an unusual appearance of his lower lip. The patient reported that the lesion initially developed in 2019 and progressed over the subsequent 6 years. He indicated that the lesion was painless and attributed its development and progression to habitual lip biting. The patient indicated that there was no family history of similar presentations. Clinical examination revealed several hard fissures and papillary projections on the lower lip (Figure 1a). Bilateral submandibular lymphadenopathy was observed, characterized by firm, tender, and mobile lymph nodes. The remainder of the intraoral and extraoral examination yielded unremarkable findings.

The patient's medical history included bronchial asthma and a recent cholera infection, for which he received advice to discontinue khat consumption. While the patient was a nonsmoker, he reported chronic khat use. Laboratory findings indicated anemia, elevated liver enzymes, and slightly elevated random blood glucose levels. A dermatologist prescribed iron supplements, multivitamins, vitamin B complex, vitamin D, folic acid, and ginseng extract. Particularly noteworthy was the patient's observation of significant lesion regression following cessation of khat use and using the above-prescribed medications (Figure 1b, taken on July 1, 2025), with recurrence upon resumption of khat consumption, suggesting a potential association between khat use and lesion activity. In response to the patient's anemia and elevated liver enzymes, a referral has been made to the Internal Medicine Department at Al-Jumhori Hospital for specialized evaluation.

## 3. Case 2

**Figure 2:**
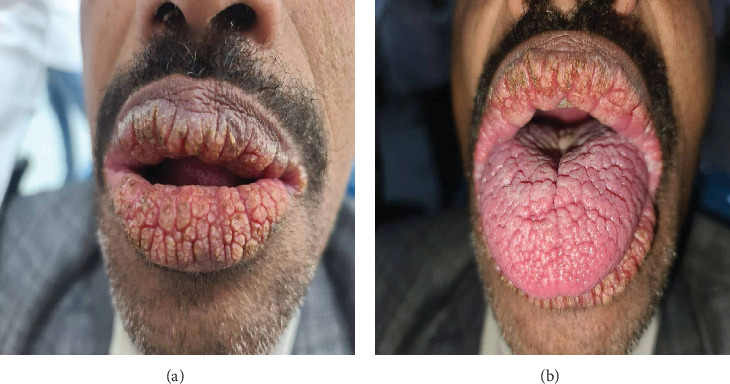
Clinical photograph illustrating Case 2 showing (a) patient exhibiting multiple papillary lesions with fissuring on the vermillion surfaces of both upper and lower lips. (b) Multiple papillary lesions on the tongue.

A 45-year-old male patient presented at Al-Razi University's Oral Medicine Outpatient Clinic in Sana'a on May 10, 2025, with complaints of xerostomia and lip discoloration. The patient reported that the lesion had initially appeared approximately 7 years prior and had exhibited gradual progression. He noted experiencing mild burning sensations associated with the lesion. This patient also denied any family history of similar clinical presentations. Despite an otherwise unremarkable medical history, laboratory investigations revealed elevated liver enzymes, alkaline phosphatase, and erythrocyte sedimentation rate (ESR). The patient's social history included significant tobacco use (40–50 cigarettes daily) and regular khat consumption. Extraoral clinical examination showed several grooves and papillary ridges on the vermillion surfaces of the upper and lower lips, including the commissures (Figure 2a). Intraoral assessment revealed that corrugation and cobblestone-like alterations were evident on the palate, labial and buccal mucosa, and tongue (Figure 2b). Notably, the patient indicated that the lesion developed coincident with his employment as a pushcart vendor, suggesting potential occupational or environmental exposure factors.

**Figure 3:**
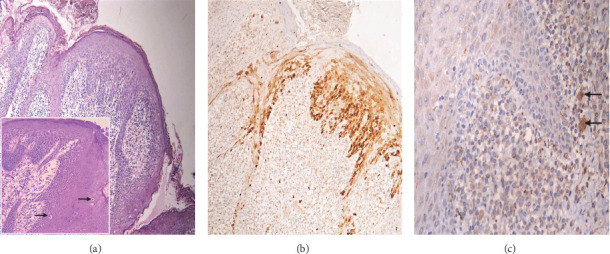
Photomicrographic of Case 1 showing (a) papillomatous growth of keratinized stratified squamous epithelium (H&E x100), the inset showing koilocytes (arrows) (H&E x200). (b) Basal and parabasal positive expression of p16 (IHC x100). (c) Negative expression of CD68 except for sporadic macrophage positivity (arrows) (IHC x400).

**Figure 4:**
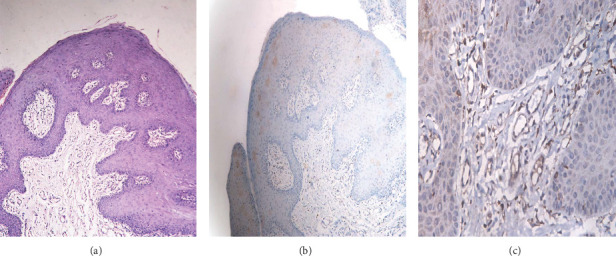
Photomicrographic of Case 2 showing (a) papillary growth of hyperplastic keratinized stratified squamous epithelium (H&E x100). (b) Negative expression of p16 (IHC x100). (c) Negative expression of CD68 (IHC x400).

**Figure 5:**
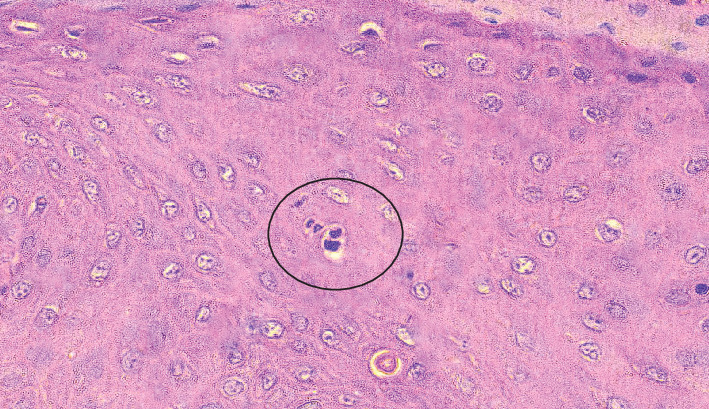
Photomicrographic showing mitosoid cells (black circle) (H&E x400).

The differential diagnosis for both cases included Heck's disease, verruciform xanthoma, oral mucosal manifestations of Crohn's disease, and Cowden's syndrome. Incisional biopsies were performed for both cases, with specimens submitted for histopathological evaluation. Histopathological examination revealed papillomatous growth of stratified squamous epithelium with hyperplasia characterized by regional parakeratosis (Figures 3a and 4a). Evidence of perinuclear vacuolization in numerous epithelial cells with pyknotic nuclei (koilocytosis) was observed (Figure 3a). Occasional mitosoid cells were also identified (Figure 5). Subepithelial chronic inflammatory cell infiltrates were present. Notably, no oral epithelial dysplasia was detected in any of the examined sections. Given the constraints regarding HPV 13, and 32 antibodies specificity and the unavailability of genetic testing infrastructure in Yemen, IHC for these viruses and PCR analysis could not be conducted. Consequently, we implemented an alternative diagnostic strategy utilizing p16 expression assessment, which exhibits established cross-reactivity, specifically with HPV 32 [22], in conjunction with CD86 evaluation to exclude verrucous xanthoma from the differential diagnosis. Regarding p16 marker evaluation, Case 1 exhibited patchy, nondiffuse nuclear positivity within the basal and parabasal epithelial cells (Figure 3b), whereas Case 2 displayed negative expression (Figure 4b). Additionally, immunohistochemical analysis using CD68 showed negative results in both cases, thereby excluding verruciform xanthoma (Figures 3c and 4c). Based on the clinical history, histopathological findings, and immunohistochemical results, a diagnosis of MEH (Heck's disease) was established.

## 4. Discussion

MEH represents a rare benign condition among HPV-related diseases. This condition demonstrates a notable predilection for children and adolescents without significant gender disparity, particularly in regions with the highest reported prevalence such as Greenland [23]. In the literature, a slight predilection for females has been reported [3]. Our study documented two male patients with this disorder, both over 30 years of age. These findings are consistent with Henke et al.'s study that has observed a mean age range between 28 and 75 years among patients in Germany [24]. In Sweden, the mean age for MEH was reported to be considerably higher, approximately 45 years and above [25]. Of note, a recent systematic review indicated a mean age of 23 years [11]. This younger mean age may be attributed to including populations with both high and low incidence of this condition.

A noteworthy aspect of these two cases was the location of the MEH lesion. The literature clearly establishes that the labial mucosa of the lower lip and buccal mucosa are the most commonly affected sites [10]. However, in both of our patients, lesions were observed on the vermillion surfaces of the lip, with the second case exhibiting multiple affected sites including the tongue and palate. We suggest that this atypical presentation can be attributed to the multifactorial nature of MEH. This condition involves viral etiology combined with genetic predisposition, which may be activated by environmental factors.

While HPV Subtypes 13 and 32 are the primary etiological factors for MEH, several predisposing factors have been identified that strongly correlate with this disorder. The most significant risk factors include socioeconomic disadvantage, inadequate oral hygiene, nutritional deficiencies, and vitamin insufficiency [8, 26, 27]. These factors align with the circumstances of our patients, as both individuals come from households with limited financial resources. Notably, both patients presented with elevated liver enzymes. Current literature does not establish a direct association between liver dysfunction and the manifestation of this condition; however, this may potentially relate to liver function affecting nutritional status and micromineral absorption [28]. Additionally, liver dysfunction exhibits a bidirectional relationship with immunity, wherein impaired liver function can compromise immune function [29]. Given that MEH is a viral infection, this relationship may help explain the presentation of this lesion in these two patients with compromised immune systems. Indeed, in support of the strong link between immune dysfunction and MEH, literature has thoroughly documented the occurrence of MEH in patients with HIV and various other immunodeficiency-related diseases [30, 31].

Of particular interest is that Case 1 demonstrated significant improvement and lesion resolution following the cessation of khat chewing. It is important to highlight that both patients shared common factors of khat chewing and elevated liver enzyme levels. This observation raises questions regarding the relationship between khat consumption, liver diseases, and MEH as a viral infection. Previous research has documented substantial adverse effects of khat on liver tissues due to its alkaloid components and the organophosphorus added during cultivation [32, 33]. Additionally, studies with animal models have established a connection between khat consumption and immune deficiency, which may facilitate viral proliferation [34, 35]. In line with these findings, recent research indicates that khat significantly increases the viral load of hepatitis B [36]. These findings suggest that khat chewing may represent a risk factor for MEH in populations where this practice is common, particularly in Yemeni communities. This evidence also helps explain the improvement observed in our first case upon discontinuing khat, while the second patient, who continued khat use, showed no clinical improvement. Even though, it remains to be determined whether khat directly affects the viruses or if it adversely impacts the liver, potentially leading to liver damage that may subsequently activate the viruses.

Clinical imaging and histopathological criteria remain the primary diagnostic standards for MEH. While advanced PCR techniques now enable the identification of HPV Types 13 and 32 commonly associated with this condition, such technology is not universally accessible due to cost constraints and infrastructure requirements, particularly in Yemen. Consequently, our investigation, alongside histopathology, focused on p16 examination, given the documented coinfection with HPV Types 16, and the documented genetic relationship between viruses associated with focal epithelial hyperplasia and HPV 16 (particularly HPV 32) [7, 22]. Our results indicated that Case 1 tested positive for this surrogate marker of HPV 16, while Case 2 showed negative results. Although HPV 16 is classified as a high-risk subtype with malignant transformation potential, current evidence suggests that its presence in MEH does not increase transformation risk. Nonetheless, it is important to note that a documented case report demonstrated that Heck's disease infection with HPV 24 progressed to malignancy, highlighting the importance of genotyping in this benign lesion [37]. Therefore, we advised the patient to maintain consistent follow-up appointments for proper monitoring.

This condition typically does not require treatment and often resolves on its own, especially in children, though various treatment options have been recommended [3]. These range from conservative approaches such as vitamin and zinc supplementation [38, 39] and topical imiquimod [40, 41] to trichloroacetic acid [42], each with varying success rates. Surgical interventions may also be appropriate in certain cases, including CO_2_ laser treatment, cauterization, cryotherapy, or surgical excision. In our first case, the patient showed significant improvement with vitamin supplementation and cessation of khat chewing, resulting in the resolution of some lesions. For the second case, the patient declined all treatment options.

Finally, the incidence of this condition in Yemen remains undocumented, as is the case in several other Middle Eastern countries. This is likely due to the rarity of the condition or insufficient research resources in the region. For instance, a limited number of case reports have been documented in Iran [43, 44], with no available documentation existing from Saudi Arabia and Jordan. To our knowledge, only one previous study has reported three cases in the Ibb city district, all of whom were younger than 15 years of age [45]. In that study, one case documented a family history involving three siblings who presented with similar symptoms. However, we did not observe this familial pattern in our two cases, as neither patient reported a family history of the condition.

## 5. Conclusion

MEH represents a rare condition in Yemen; however, ongoing socioeconomic challenges and regional instability, where chewing khat is a common daily practice, may contribute to an increased incidence. Dental practitioners should consider including this condition in their differential diagnosis when evaluating multiple lesions affecting the oral cavity or the vermillion surfaces of the lips.

## Data Availability

The data supporting the finding of this study is available within the manuscript.
